# Supplementary material for: Three-Fluorophore FRET Enables the Analysis of Ternary Protein Association in Living Plant Cells
Source: Plants (Basel). 2022 Oct 6;11(19):2630. doi: 10.3390/plants11192630 (PMC9571070; doi:10.3390/plants11192630)
Supplement: Supplementary file 1 [file plants-11-02630-s001.zip › plants-1927475-supplementary.pdf]

Three-fluorophore FRET enables the analysis of ternary protein association in living plant cells

Nina Glöckner[a], Sven zur Oven-Krockhaus[a,b], Leander Rohr[a], Frank Wackenhut[b], Moritz Burmeister[b], Friederike Wanke[a], Eleonore Holzwart[c], Alfred J. Meixner[b], Sebastian Wolf[c] and Klaus Harter[a*]

[a]Center for Plant Molecular Biology, University of Tübingen, Tübingen, Germany
[b]Institute for Physical & Theoretical Chemistry, University of Tübingen, Tübingen, Germany
[c]Centre for Organismal Studies, University of Heidelberg, Heidelberg, Germany

*corresponding author (Email: klaus.harter@zmbp.uni-tuebingen.de)

Supplementary Materials

Supplemental Notes......2

Theoretical background......2

Calculations for FRET cascades......3

Cross-talk during imaging......5

Process of spectral unmixing based on λ-stacks......6

Supplementary Tables......7

Table S1. Parameters of fluorophores in this study......7

Table S2. Coefficients for spectral bleed-through (bt) and cross-excitation (ce)......7

Table S3. List of primers......8

Supplementary Figures......9

Figure S1. RLP44-mTRQ2, BRI1-mVEN and BAK1-mRFP are expressed together N. benthamiana epidermal leaf cells......9

Figure S2. Wavelength-dependent intensity measurements reveal different fusion protein expression levels for different plasma membrane regions in transiently transformed N. benthamiana epidermal leaf cells......11

Figure S3. BRI1^{HA} is expressed in transiently transformed N. benthamiana leaf cells......12

Figure S4. FLS2 does not interfere with the RLP44-related interactions (extension to Fig. 4)......13

Figure S5. The overall donor to acceptor ratios is not significantly different in the protein fusion arrangements of the FRET-FLIM analysis......15

Figure S6. Example of spectral unmixing process based on λ-stacks......16

References......17

Supplemental Notes

Theoretical background

The FRET efficiency (E) between a donor (D) and an acceptor (A) fluorophore is given by

$$E = \frac{R_0^6}{R_0^6 + r^6}, \quad (1)$$

with r as the distance between donor and acceptor and R_0 as the distance for 50 % energy transfer (Förster distance):

$$R_0\ (nm) = 0.02108 \cdot (\kappa^2 \cdot n^{-4} \cdot QY_D \cdot J)^{1/6}, \quad (2)$$

with κ^2 as the dipole orientation factor, n as the refractive index of the medium, QY_D as the quantum yield of the donor and J as the spectral overlap between donor emission and acceptor absorption:

$$J = \varepsilon_A \frac{\int f_D(\lambda) f_A(\lambda) \lambda^4 d\lambda}{\int f_D(\lambda)\, d\lambda}, \quad (3)$$

with ε_A as the molar attenuation coefficient of the acceptor at the peak absorption wavelength, λ as the wavelength in nm, f_D and f_A as the normalized donor emission and acceptor absorption spectra, respectively.

As evident in equation (2), the dipole orientation factor κ^2 has a strong influence on the calculated R_0 and thus E:

$$\kappa^2 = cos^2\, \omega\ (1 + 3cos^2\theta). \quad (4)$$

Here, ω is the angle between the electric field vector of D at the location of A and the absorption dipole orientation of A and θ is the angle between the emission dipole of D and the separation vector of D-A (Lakowicz 2006).

For the standard assumption of κ^2 = 2/3 to be true, the rotational diffusion of a fluorophore has to be faster than the fluorescence lifetime (FLT) of D (Müller et al. 2013; Hink et al. 2002). However, this may not be true for genetically-encoded fluorescent proteins used in FRET studies for several reasons: (i) The fluorophore barrel is large and has a rotational correlation time of about 20–30 ns, whereas the FLT is in a range of 1–4 ns (Vogel et al., 2012). (ii) The fluorophores are attached to the proteins of interest with a flexible linker (Chen et al. 2013; van Rosmalen et al. 2017; George and Heringa 2002; Chen et al. 2013). In previous studies, a linker length of 15 amino acids was assumed to allow free rotation of the fluorophore, even though this may not be fully true (Ujlaky-Nagy et al. 2018; Szöllosi et al. 2006; Shrestha et al.

201). But as no better options are available, the standard assumption is used. This introduces an error due to different fluorophore orientations. Hink et al. (2002) proposed to use κ^2 = 0.476, which is the value of the orientation factor for a rigid, randomized ensemble of D-A pairs (Steinberg 1971). This effectively reduces the calculated Förster distance. It does not eliminate the possibility of specifically existent deviations due to fixed protein arrangements, e.g., preventing FRET or other spatial arrangements. It is important to keep in mind that the FRET efficiency does not correspond to fixed real distances (Müller et al. 2013): The presence of FRET always means that they are in close proximity, but how close exactly can rarely be precisely determined, especially in live-cell imaging. The distance r that is accessible through FRET-measurements is in average between 0.5 $R_0 \leq r \leq 1.5\, R_0$ (Gadella 2009; Müller et al. 2013). Most FRET pairs have Förster distances between 4 and 7 ns (Bajar et al. 2016; Mastop et al. 2017). As a rule of thumb, FRET is restricted to distances below 10 nm. The absence of FRET does not necessarily mean that the proteins of interest are not in close proximity or do not interact, e.g., due to unfavorable fluorophore attachment positions. Three-fluorophore FRET-FLIM, in turn, has the same limitations.

Calculations for FRET cascades

We define a cascading, linear three-chromophore FRET system

1 — 2 — 3

with 3 as the most redshifted fluorophore. When 2 acts as the donor, the spectral overlap of 2's emission spectrum with 1's absorption spectrum is negligible. The FRET efficiency for the path 2 ⟶ 3 can, therefore, be calculated with (1) as follows:

$$E_{23} = \frac{R_{0\,23}^6}{R_{0\,23}^6 + {r^6}_{23}}. \tag{5}$$

When 1 acts as the donor, two paths are possible:

1 ⟶ 2 ⟶ 3 (energy transfer to 3 via 2) or

1 ⟶ 3 (direct energy transfer to 3).

The energy can follow either path, so the energy transfer possibility for 1 ⟶ 2 is diminished by the energy transfer possibility for 1 ⟶ 3 and vice versa. This decrease can be expressed in terms of the fluorophore's quantum yield, using the following equations by Liu & Lu (2002). For 1 ⟶ 2:

$$\Delta\mathrm{QY}_{12} = \frac{R_{0_{13}}^{6}}{R_{0_{13}}^{6} + r^{6}{}_{13}} \tag{6}$$

R_0 to the power of 6 contains QY as a multiplicator (cf. (2)), so the lost fraction of QY can be implemented in R_0 as follows:

$$R_{0_{12}}^{6}{}' = R_{0_{12}}^{6} \cdot (1 - \Delta\mathrm{QY}_{12}) = R_{0_{12}}^{6} \cdot \left(1 - \frac{R_{0_{13}}^{6}}{R_{0_{13}}^{6} + r^{6}{}_{13}}\right), \tag{7}$$

with $R_{0_{12}}^{6}{}'$ and $R_{0_{12}}^{6}$ as the Förster distance of 1 ⟶ 2, when 3 is present or absent, respectively.

This adjusted Förster distance now replaces the numerator in (1):

$$E_{12} = \frac{R_{0_{12}}^{6}{}'}{R_{0_{12}}^{6} + r^{6}{}_{12}} \tag{8}$$

Combining and simplifying (6) and (7):

$$E_{12} = \frac{\left(R_{0_{12}} \cdot r_{13}\right)^{6}}{\left(R_{0_{12}} \cdot r_{13}\right)^{6} + \left(R_{0_{13}} \cdot r_{12}\right)^{6} + (r_{12} \cdot r_{13})^{6}} \tag{9}$$

A similar result can be acquired for 1 ⟶ 3:

$$E_{13} = \frac{\left(R_{0_{13}} \cdot r_{12}\right)^{6}}{\left(R_{0_{13}} \cdot r_{12}\right)^{6} + \left(R_{0_{12}} \cdot r_{13}\right)^{6} + (r_{12} \cdot r_{13})^{6}} \tag{10}$$

The overall FRET efficiency for the above 1 ⟶ 2 ⟶ 3 FRET cascade can now be estimated, using (5), (9) and (10):

$$E_{casc.} = E_{12} \cdot E_{23} + E_{13} \tag{11}$$

We acknowledge that this linear FRET cascade approach neglects, e.g., the arrangement 2 — 1 — 3. The truth will be much more complicated, including complex geometric arrangements of the fluorophores, enabling a multitude of possible energy transfer pathways that will not be solved easily without performing extensive numerical simulations. However, this set of equations and the derived results illustrate how a third fluorophore changes the FRET working parameters and how important it is to consider the actual biological situation – in this example, the analysis of protein complexes in the

plasma membrane, which is of great common interest in the life science community. Although these theoretical considerations can only be simplified approximations, they impressively show that FRET interaction assays can, under certain conditions, cover much larger distances than generally expected, making this method viable for a much broader range of analyses.

Cross-talk during imaging

Cross-talk in the form of spectral bleed through (bt) and cross-excitation (ce) was present for our fluorophores. To account for this, the bt and ce was assessed from normalized absorption and emission spectra and quantified by imaging single-fluorophore expressing *N. benthamiana* plants.

Excitation at 458 nm lead to a relative absorption of 91% for mTRQ2 and a cross-excitation of 8% for mVEN and 3% for mRFP (Fig. 1A purple, vertical line). Excitation at 514 nm for mVEN yielded a relative absorption of 99% and a cross-excitation of mRFP of 20% (Fig. 1A green line). mRFP was excited with 561 nm with a relative absorption of 61% (Fig. 1A orange line). Spectral bleed-through was present from mTRQ2 to mVEN and from mVEN to mRFP: When detecting mVEN with a bandwidth from 525 to 565 nm, 35% of the signal originates from mTRQ2 (Fig. S2, Table S2). Detection of mRFP between 605 and 650 nm included 6% of mVEN emission (Fig. S2, Table S2). When quantifying the FI, also the molecular brightness of a fluorophore, detection settings such as detector gain and laser strength are influencing the amount of bleed-through and cross-talk. Thus, we calculated the coefficients from imaging single-labelled samples with sequential excitation (Table S2). Measured bt and ce from mTRQ2 to the yellow and red channel was higher than calculated, as higher laser settings were used to excite mTRQ2. Measured ce from mVEN to the red channel was in the same order of magnitude as calculated. As the molecular brightness of mVEN is relatively high (Table S1), the bt to the red channel was higher than calculated (Table S2), even with reduced laser power for 514 nm compared to the 458 nm and 561 nm laser lines (see Material and methods).

Process of spectral unmixing based on λ-stacks

For λ-stacks, the emission of the sample is recorded in several narrow spectral regions one after the other. Figure S 6b shows such a λ-stack with an excitation wavelength of 458 nm comprising 21 images, each assigned to a spectral region (here with a spectral width of 8 nm). The total detected intensity over all wavelength ranges can be obtained by summing the intensities per pixel along the λ-stack, resulting in an overview image as shown in Figure 6a. Using such an overview image, regions of interest (ROIs) can now be selected. All pixel intensities within a ROI are summed per wavelength range to extract the corresponding spectra (Fig. S6c), where each wavelength range is represented by its central wavelength.

Linear spectral unmixing aims to find a linear combination of the component spectra of mTRQ2, mVEN, and mRFP that best approximates the measured spectrum, pointing to the relative fractions of the fluorophores that were present in the sample. An exemplary graphical representation of the unmixing result can be found in Figure S6d. FRET efficiency calculations can be based on these fractions according to the formulas above, also considering cross-excitation and spectral bleed-through. MATLAB scripts for the corresponding calculations are available at https://github.com/svenzok/3F-FRET. Spectral unmixing with ROIs is based on low pixel numbers and/or intensities. Accordingly, for statistically robust results, great care must be taken to randomize ROI selection and to analyze a reasonable number of biological replicates.

Supplementary Tables

Table S1. Parameters of fluorophores in this study

Quantum yield (QY), Molar attenuation coefficient (ε) at the absorbance maximum, Brightness as the product QY·ε, Maturation half-time at 37°C in E. coli (t_{50}), Photostability as half-time of bleaching under laser scanning illumination with 80 µW ($t_{½}$), pH stability (pK_a) and proportion of monomeric state. For comparison: EGFP features a brightness of 33.6 $mM^{-1}cm^{-1}$, a bleaching half-time of 159.7 s and a pK_a of 6.0.

	QY	ε [mM^{-1} cm^{-1}]	Brightness [mM^{-1} cm^{-1}]	Maturation half-time t_{50} [min]	Photostability $t_{½}$ [s]	pH stability pK_a	monomer [%]
mTRQ2	0.93	30	27.9	33.5	71.7	3.1	93.8
	(Goedhart et al. 2012; Cranfill et al. 2016; Balleza et al. 2018)						
mVEN	0.64	105	67.2	17.6	26.5	5.5	83.9
	(Kremers et al. 2006; Cranfill et al. 2016; Balleza et al. 2018)						
mRFP	0.25	44	11.0	21.9	26.3	4.5	95.8
	(Campbell et al. 2002; Cranfill et al. 2016; Balleza et al. 2018)						

Table S2. Coefficients for spectral bleed-through (bt) and cross-excitation (ce)

The bt and ce coefficients with standard error (SE) for relevant laser lines were both measured in images when only the relevant fluorophore was expressed (D:A1:A2) and additionally calculated based on normalized spectra.

	bleed-through (bt)			cross-excitation (ce)		
	1 >> 2	1 >> 3	2 >> 3	1 >> 2	1 >> 3	2 >> 3
Average false/true signal	0.41	0.04	0.14	0.11	0.03	0.16
SE	0.01	0.001	0.02	0.01	0.01	0.01
Ratio D:A1:A2	1:0:0	1:0:0	0:1:0	0:1:0	0:0:1	0:0:1
Excitation laser [nm]	458	458	514	458	458	514
Calculated false/true signal	0.35	0.01	0.06	0.08	0.03	0.2

Table S3. List of primers.

GOI – vector	Forward primer 5‘ » 3‘	Reverse primer 5‘ » 3‘
RLP44 - pDONR221-P3P2	GGGGACAACTTTGTATAATAAAGTTGtaATGACAAGGAGTCACCGGTTAC	GGGGACCACTTTGTACAAGAAAGCTGGGTtGTAATCAGGCATAGATTGAC
BRI1 - pDONR221-P1P4	GGGGACAAGTTTGTACAAAAAAGCAGGCTtaATGAAGACTTTTTCAAGCTTCTT	GGGGACAACTTTGTATAGAAAAGTTGGGTGTAATTTTCCTTCAGGAACTTCTT
FLS2 - pDONR221-P1P4	GGGGACAAGTTTGTACAAAAAAGCAGGCTtaATGAAGTTACTCTCAAAGAC	GGGGACAACTTTGTATAGAAAAGTTGGGTGAACTTCTCGATCCTCGTTACG
FLS2 - pDONR207	GGGGACAAGTTTGTACAAAAAAGCAGGCTtaATGAAGTTACT	GGGGACCACTTTGTACAAGAAAGCTGGGTgAACTTCTCGATCCT

Supplementary Figures

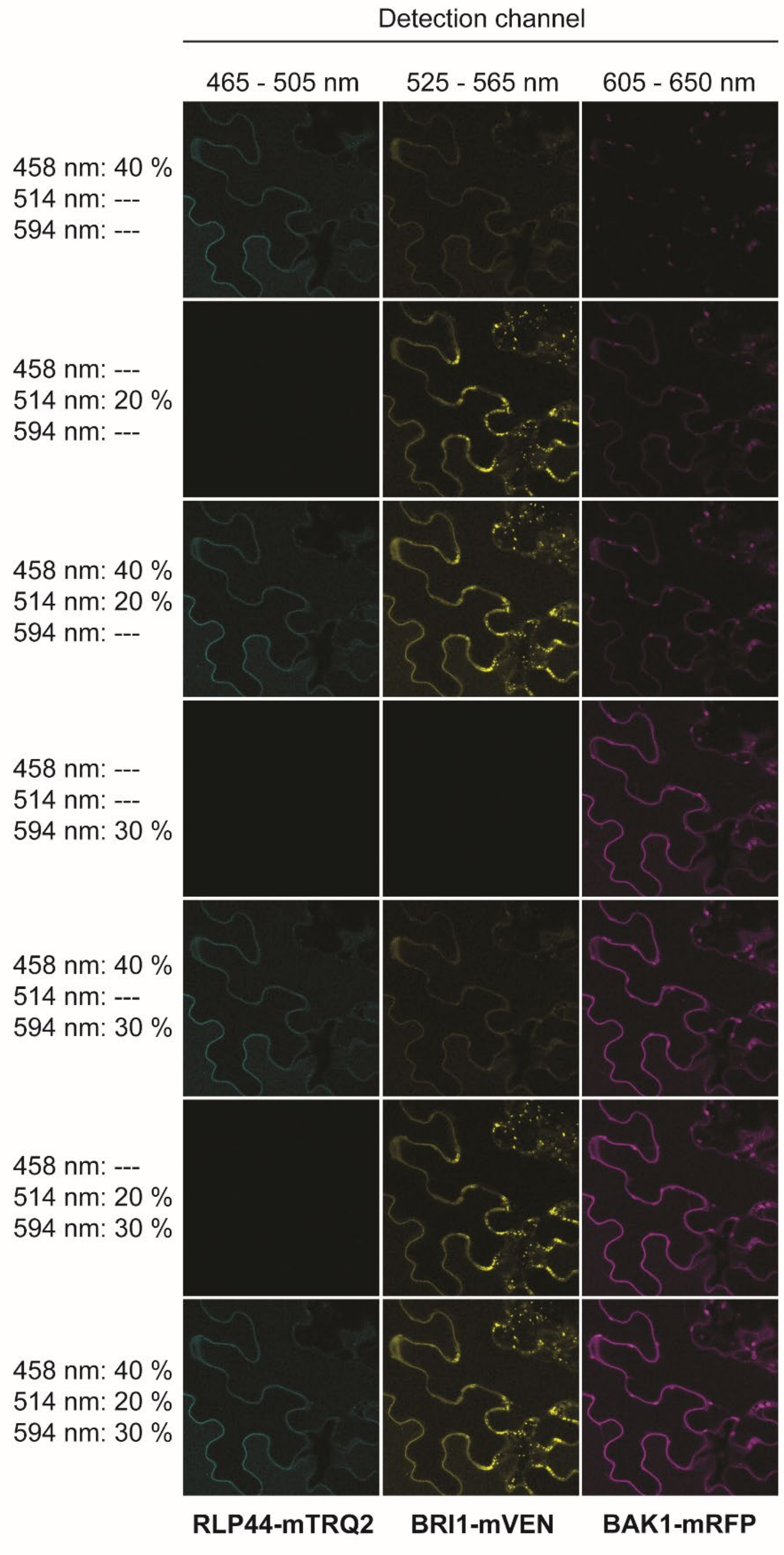

Figure S1. RLP44-mTRQ2, BRI1-mVEN and BAK1-mRFP are expressed together **in** *N. benthamiana* epidermal leaf cells.

Representative confocal images of the fluorescence intensity in the mTRQ2 channel, the mVEN channel and the mRFP channel are exemplary shown for the combination of RLP44-mTRQ2, BRI1-mVEN and BAK1-mRFP two days after transient transformation of *N. benthamiana* leaf cells. The excitation wavelengths and the relative laser intensities are depicted to the left. The blue channel for detection of RLP44-mTRQ was 465-505 nm, the yellow channel for detection of BRI1-mVEN was 525-565 nm and the red channel for detection of mRFP was 605-650 nm. The scale bar represents 20 µm. In general, before the acquisition of spectra or the fluorescence intensities, the expression of each fusion construct was determined by sequential excitation, meaning that one laser line was switched on at a time.

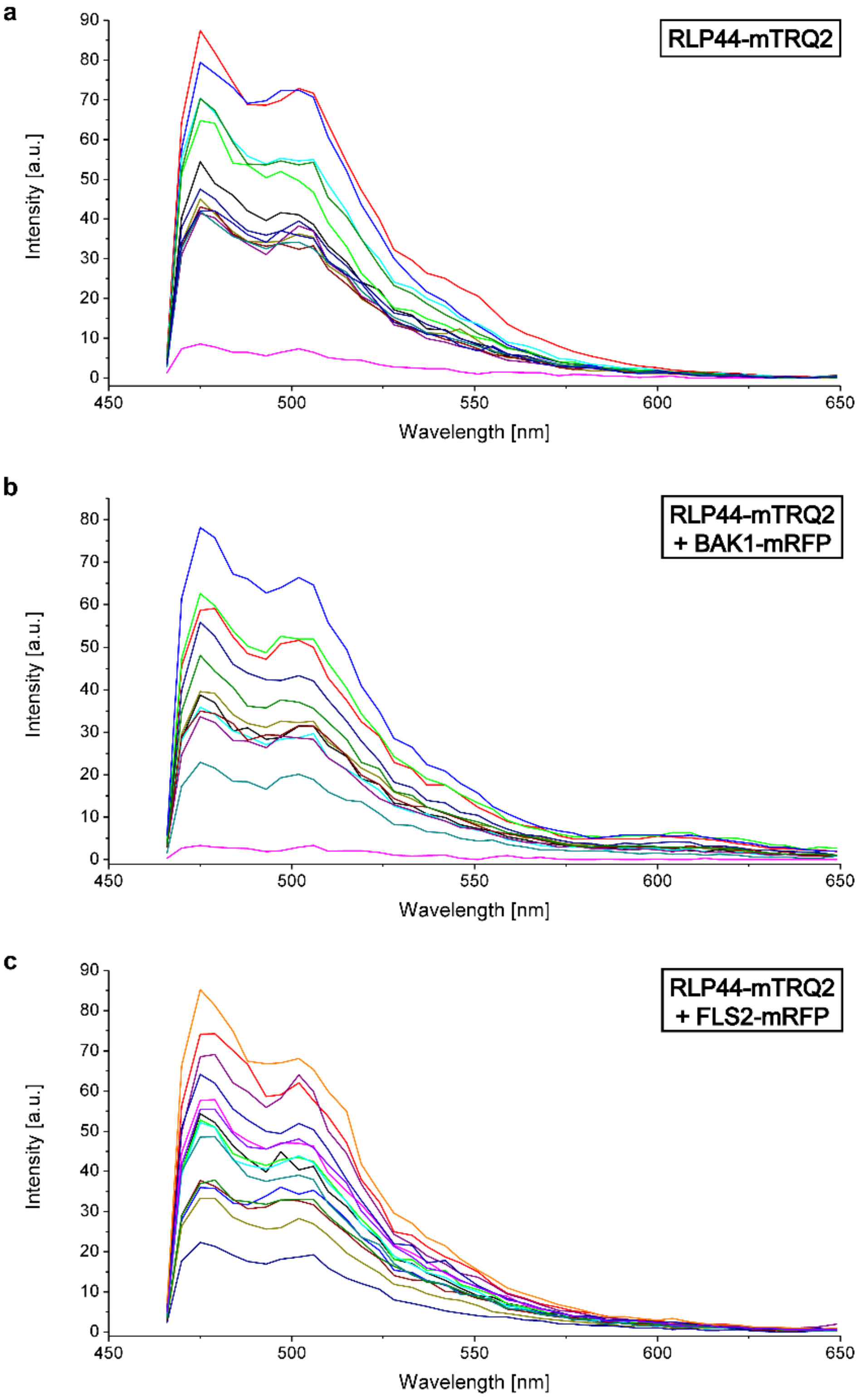

Figure S2. Wavelength-dependent intensity measurements reveal different fusion protein expression levels for different plasma membrane regions in transiently transformed N. benthamiana epidermal leaf cells.

The wavelength-dependent raw intensity of 13 different regions of interest, each, in the plasma membrane of *N. benthamiana* leaf cells after excitation with light of 458 nm is depicted for RLP44-mTRQ2 alone (**a**), the RLP44-mTRQ2 + FLS2-mRFP (**b**) and RLP44-mTRQ2 + FLS2-mRFP pair (**c**).

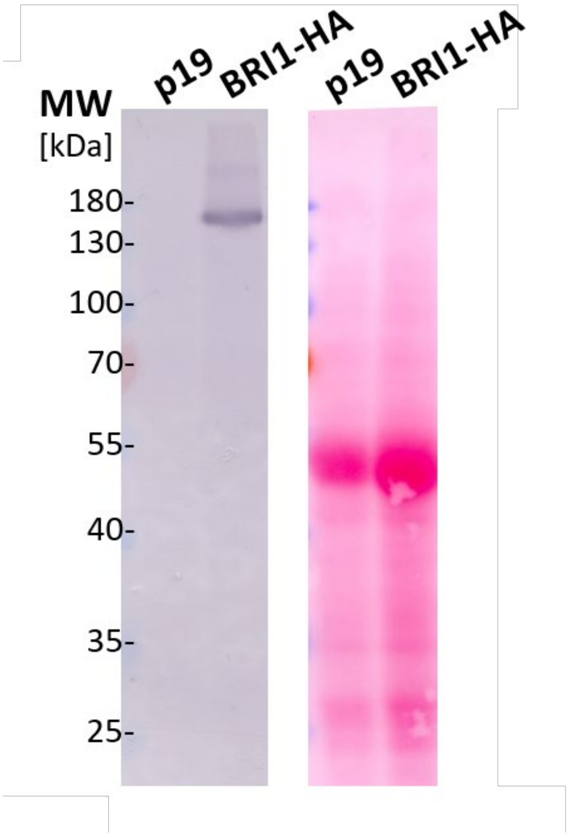

Figure S3. BRI1HA is expressed in transiently transformed N. benthamiana leaf cells.

Western blot (left) and the total protein on the transfer membrane, stained with Ponceau S (right) of *N. benthamiana* leaf extracts prepared two days after transformation with either the silencing inhibitor plasmid p19 (control) or with p19 and a plasmid coding for BRI1^{HA}. BRI1^{HA} was detected with an HA antibody. The protein size markers are shown to the left.

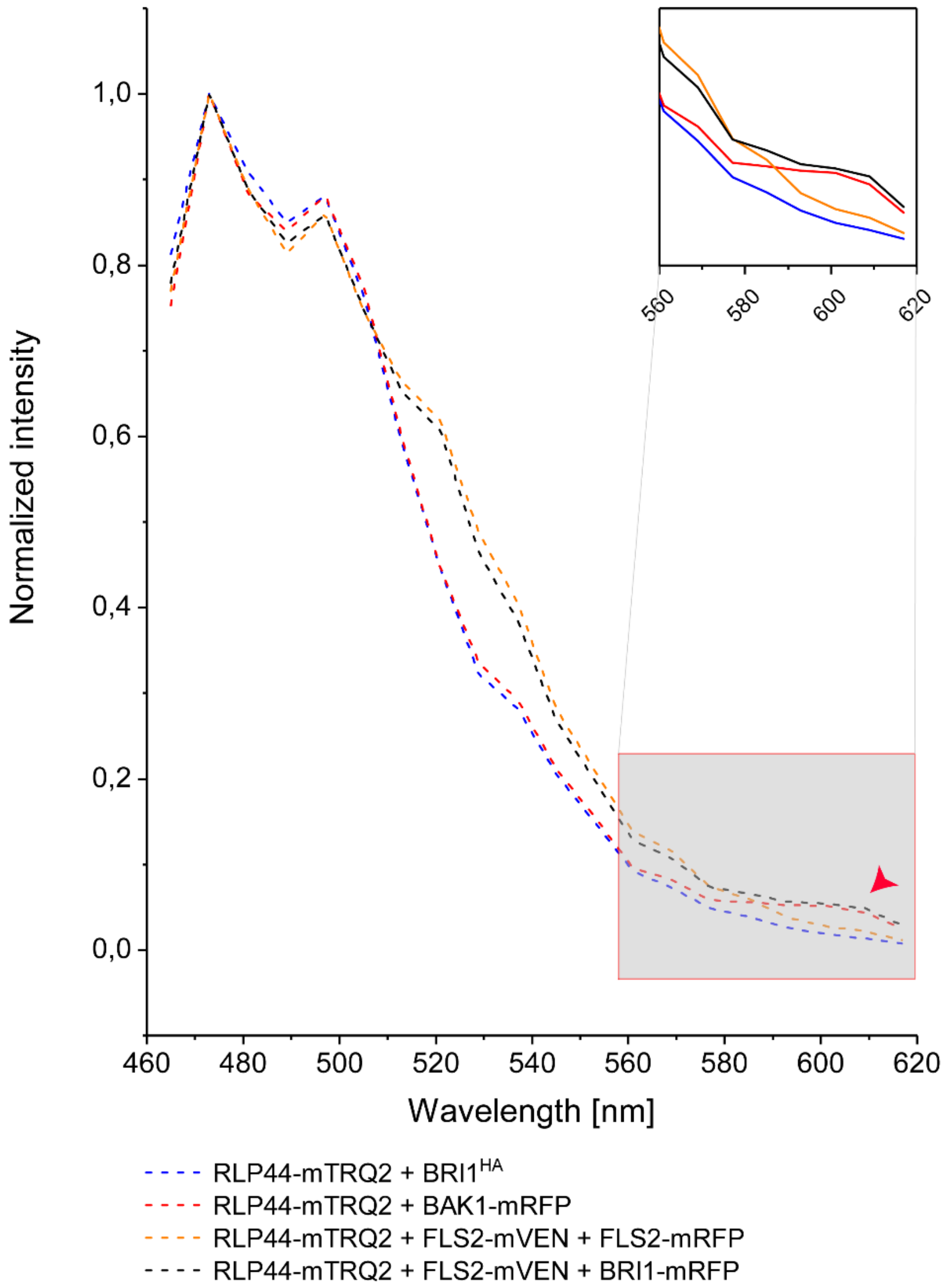

Figure S4. FLS2 does not interfere with the RLP44-related interactions (extension to Fig. 4). Wavelength-dependent normalized fluorescence emission after irradiation of the *N. benthamiana* leaf cells with 485 nm light for the co-expression of RLP44-mTRQ2 with BRI1^{HA} (blue) or with BAK1-mRFP (red) or FLS2-mVEN and FLS2-mRFP (orange) or FLS2-mVEN and BRI1-mRFP4 (black). The FRET-relevant wavelength area is highlighted in the enlarged section. The occurrence of FRET from mTRQ2 to mRFP is indicated by a red arrow head.

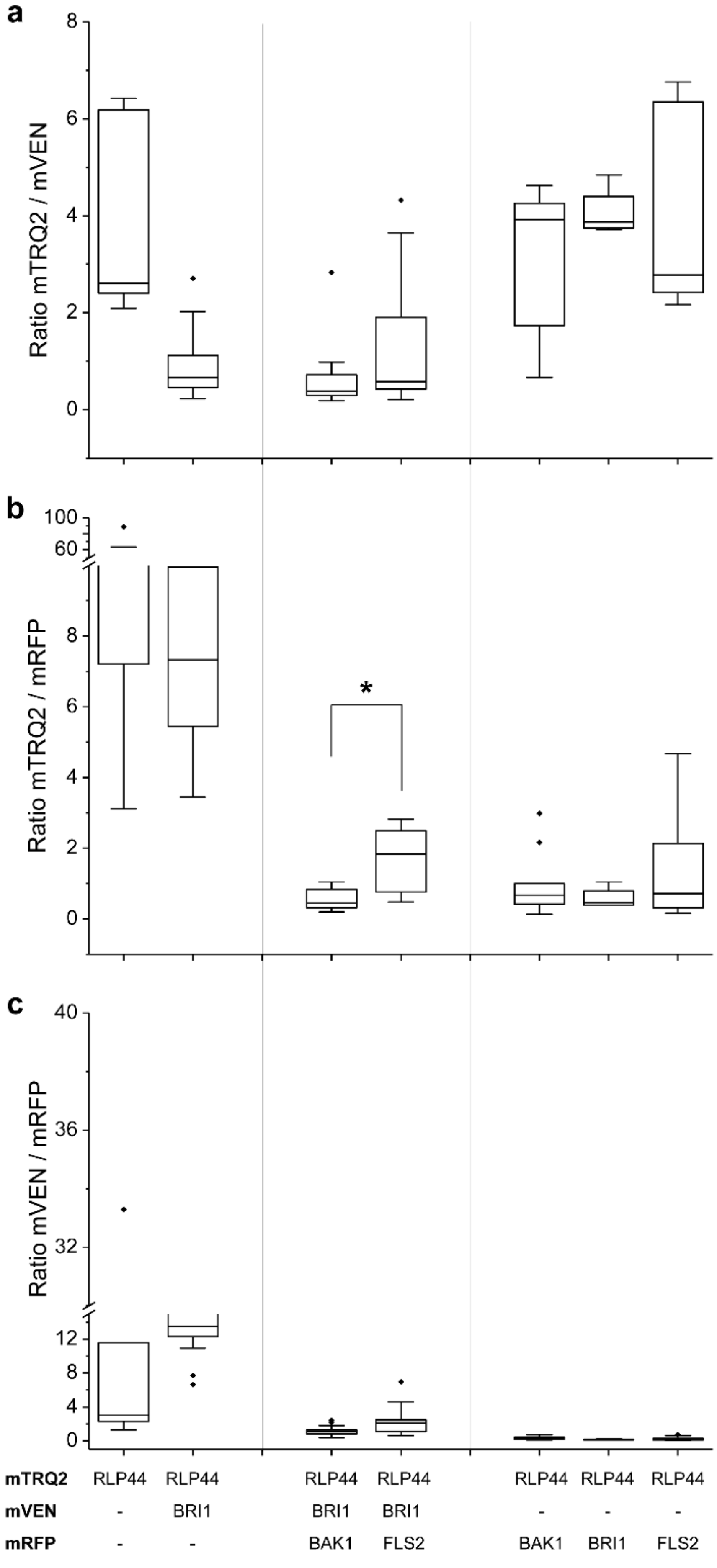

a
b
c
Ratio mTRQ2 / mVEN
Ratio mTRQ2 / mRFP
Ratio mVEN / mRFP
*
mTRQ2
mVEN
mRFP
RLP44
BRI1
BAK1
FLS2

Figure S5. The overall donor to acceptor ratios is not significantly different in the protein fusion arrangements of the FRET-FLIM analysis.

Fluorescence emission ratios for the mTRQ2/mVEN fusion protein pairs (**a**), the mTRQ2/mRFP fusion protein pairs (**b**) and mVEN/mRFP fusion protein pairs (**c**) are shown. Data points were left-right scattered (*black dots*) and combined with boxplot information, permitting outliers.

The ratios for mTRQ2/mVEN, mTRQ2/mRFP and mVEN/mRFP were tested for significant differences with a two-tailed, all-pair Kruskal-Wallis test followed by a Steel-Dwass *post hoc* correction. The asterisks marks a significant difference ($p < 0.05$) in the donor-to-acceptor ratios, which was the mTRQ2/mRFP ratio in the RLP44-mRFP4/BRI1-mVEN/BAK1-mRFP and the RLP44-mTRQ2/BRI1-mVEN/FLS2-mRFP arrangements. The boxplots represent all data with the median as a solid line within the box that is restricted by the first quartile (25 %; lower end) and the third quartile (75 %; upper end). The whiskers show the minimum and maximum value of the data, respectively, that are not defined as outliers (1.5 times the interquartile range). Outliers are indicated as black diamonds.

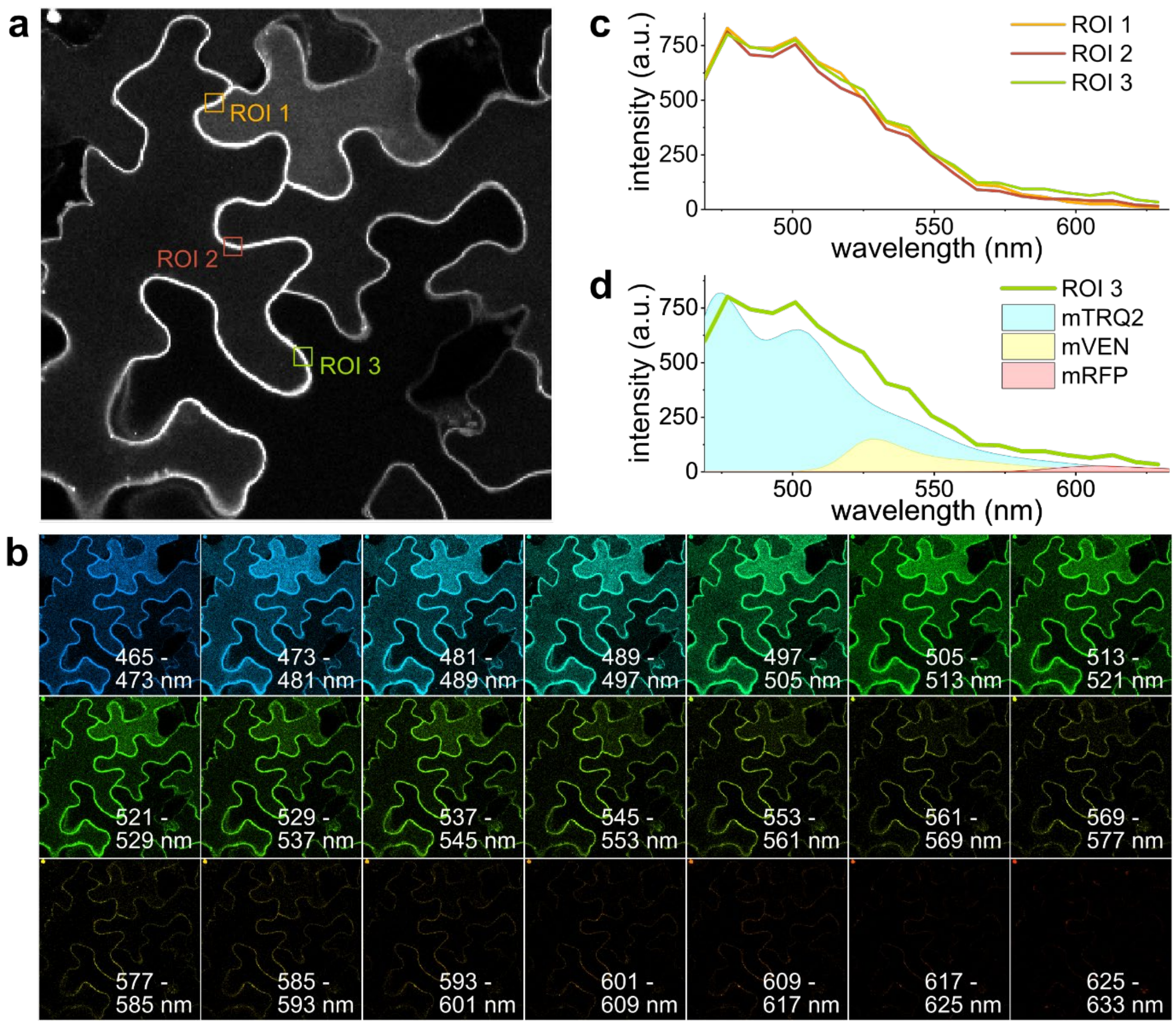

Figure S6. Example of spectral unmixing process based on λ-stacks.

Overview image **(a)** with randomly selected regions of interest (ROI 1-3) of the plasma membrane for cells expressing RLP44-mTRQ2, BAK1-mVEN and BRI1-mRFP. Each frame of the λ-stack **(b)** corresponds to an emission wavelength range, imaged consecutively under constant 458 nm excitation. Intensity summation over all wavelength ranges results in image **(a)**. Spectra **(c)** for each ROI are extracted by summing all pixel intensities of the specified ROI for each wavelength range, resulting in 21 discrete values for each ROI. The x-axis represents the respective center of each wavelength range. Spectral unmixing **(d)** is shown as a graphical representation for ROI 3 (green line). Unmixing describes the process of finding a linear combination of normalized mTRQ2 (blue), mVEN (yellow) and mRFP (red) spectra that best approximates the measured data.

References

Balleza, E., Kim, J. M., & Cluzel, P. (2018). Systematic characterization of maturation time of fluorescent proteins in living cells. *Nature Methods*, *15*(1), 47–51. https://doi.org/10.1038/nmeth.4509.

Bunt, G. & Wouters, F. S. (2017). FRET from single to multiplexed signaling events. *Biophysical reviews 9***,** 119–129; 10.1007/s12551-017-0252-z.

Campbell, R. E., Tour, O., Palmer, A. E., Steinbach, P. A., Baird, G. S., Zacharias, D. A., & Tsien, R. Y. (2002). A monomeric red fluorescent protein. *Proceedings of the National Academy of Sciences of the United States of America*, *99*(12), 7877–7882. https://doi.org/10.1073/pnas.082243699

Cranfill, P. J., Sell, B. R., Baird, M. A., Allen, J. R., Lavagnino, Z., Gruiter, H. M. de, . . . Piston, D. W. (2016). Quantitative assessment of fluorescent proteins. *Nature Methods*, *13*(7), 557–562. https://doi.org/10.1038/nmeth.3891

Goedhart, J., Stetten, D. v., Noirclerc-Savoye, M., Lelimousin, M., Joosen, L., Hink, M. A., . . . Royant, A. (2012). Structure-guided evolution of cyan fluorescent proteins towards a quantum yield of 93%. *Nature Communications*, *3*, 751. https://doi.org/10.1038/ncomms1738

Koushik, S. V., Blank, P. S. & Vogel, S. S. (2009). Anomalous surplus energy transfer observed with multiple FRET acceptors. *PloS One 4***,** e8031; 10.1371/journal.pone.0008031.

Kremers, G.-J., Goedhart, J., van Munster, E. B., & Gadella, T. W. J., JR. (2006). Cyan and yellow super fluorescent proteins with improved brightness, protein folding, and FRET Forster radius. *Biochemistry*, *45*(21), 6570–6580. https://doi.org/10.1021/bi0516273.

Liu, J., Lu, Y. (2002). FRET study of a trifluorophore-labeled DNAzyme. *J. Am. Chem. Soc., 124*(51), 15208-15216. https://pubs.acs.org/doi/10.1021/ja027647z.

Nagai, T., Ibata, K., Park, E. S., Kubota, M., Mikoshiba, K., & Miyawaki, A. (2002). A variant of yellow fluorescent protein with fast and efficient maturation for cell-biological applications. *Nature Biotechnology*, *20*(1), 87–90. https://doi.org/10.1038/nbt0102-87

Scott, B. L., & Hoppe, A. D. (2015). Optimizing fluorescent protein trios for 3-Way FRET imaging of protein interactions in living cells. *Scientific Reports*, *5*, 10270 EP -. https://doi.org/10.1038/srep10270
